# Report on Surgery for the Fifth Congressional District

**Published:** 1885-12

**Authors:** R. H. Taylor

**Affiliations:** Griffin, Ga.


### REPORT ON SURGERY FOR THE FIFTH CONGRESSIONAL DISTRICT.

BY R. H. TAYLOR, M. D., GRIFFIN, GA.

REPORTED TO THE THIRTY-SIXTH ANNUAL SESSION OF THE MEDICAL ASSOCIATION OF GEORGIA, HELD IN SAVANNAH, APRIL 15, 1885,

On the 30th day of November I was consulted by Mr. ——— in regard to his child, 15 months old, who had a "watermelon seed in its windpipe." I saw the child the following day and found her condition as follows—with this history: Some time about the first of last August, the child was suddenly seized with a fit of strangulation, while in the care of a negro nurse, who was eating some watermelon. The negro said that the child was eating with her and choked on a seed. Everything was done by the mother that she could do and with no relief. She, the mother, says from that day until now this "rattling in the throat" has continued without intermission; said she had only given some "cough drops" prepared by a neighboring physician.

About two weeks before I was consulted, the child had been carried to a physician in this place, who examined it, and said the cough was produced by an elongation of the uvula; accordingly he clipped the uvula. Still the symptoms did not abate, but continued to increase. I examined the child and found the pulse 145, temperature 104 4-5, respiration 40, and considerably labored. There were very decided mucus rales all through the bronchi on both sides, as well as an accumulation of mucus, or something I was not certain what, in the trachea. The child was very irritable and refractory, and at that age, a satisfactory examination was almost impossible. I therefore administered a full dose of dovers powder and remained with the child all night. When fully under the influence of the dovers powder, I found the respiration some better and less labored; still I could not locate any foreign body in the trachea. I so informed the parents, and told them that in the present condition of the child I would not advise an operation, at least, until I had tried to abate the urgent constitutional symptoms by general treatment, but if a sudden suffocation should threaten immediate death I would, of course, open the trachea at once. I accordingly directed the following treatment: Tinct. verat. vir.—(Norwood), spts. nit., ether and simple syrup squills every two or three hours, according to the effect on the pulse, and a full dovers powder *pro re nata*. On the next morning I saw the case again with my friend Dr. Drewry, found the pulse 120, temperature 103, respiration same. We were still unable to locate a foreign body, and Dr. D. concurred in the opinion that it was best not to interfere, unless sudden suffocation should demand immediate operation. The case was closely watched and the treatment continued until the 11th day of December. Temperature and pulse were both more favorable without any improvement in respiration. The night previous the suffocation was alarming and was still much labored. We, therefore, told the parents that we were of opinion, from the history of the case, that there was some foreign body present, although we could not locate it, but that death was the inevitable result without operative interference, and further more, the result of an operation with so much general disturbance was by no means flattering, and might fail to discover the foreign body still it was our opinion that this was all that remained to be done. With this statement they consented to the operation, and we did the ordinary inferior tracheotomy. As soon as the trachea was opened, with the violent spasmodic cough which followed the rushing in of the cold air, was expelled through the opening about two ounces of very offensive pus, mixed with a considerable quantity of shreddy mucus. The respiration was so much relieved by this that when the mother was admitted, soon afterwards, she remarked that she had not seen her baby breathe so easily since last August. A thorough search and careful exploration of the trachea with probe and finger failed to discover any foreign body.

The wound was left open, and light carbolized dressings applied. A considerable quantity of pus was expelled through the wound for several days, and the child steadily improved. The next day after the operation I discovered a cavity in the left lung, about opposite the fourth rib. The after-treatment consisted chiefly of milk and brandy, with occasionally an egg-nog, and as soon as the stomach could tolerate it, cod liver oil. Dovers powder was given in sufficient quantity to keep the little patient quiet. The restoration was slow, but steady and uninterrupted, and on the eighteenth day of January, 1885, I dismissed my little patient, who has been increasing in weight every day, and is now in as good health as the average child. I would not forget here to acknowledge my obligation to my friend, Dr. D., for his very efficient assistance, not only in the operation itself, but in the management of the case throughout.

While I am still in some doubt as to the exact nature of the foreign body, I am convinced that one was present. It could not have been a seed, as the family supposed, I do not think, without being detected in its escape, and that it did escape, I think the result proves conclusively. My opinion, however, is that it was the *pulp* of the melon, and that it was impacted in some of the smaller or minute ramifications of the bronchial tubes, producing an enfarction, by its mechanical presence caused the formation of an abcess, which ruptured at the time of the operation, leaving the cavity before mentioned. This pulp might have been discharged with the pus in shred-like particles, and escaped our notice, or it might have been what we thought at the time, tough shreds of mucus, and allowed to pass without careful examination. This, is mere supposition.

